# Matching patients to an intervention for back pain: classifying patients using a latent class approach

**DOI:** 10.1111/jep.12115

**Published:** 2014-03-24

**Authors:** Martine J Barons, Frances E Griffiths, Nick Parsons, Anca Alba, Margaret Thorogood, Graham F Medley, Sarah E Lamb

**Affiliations:** 1 Research Fellow, Complexity Science Centre, University of WarwickCoventry, UK; 2 Professor of Medicine in Society; 3 Statistician; 4 Research Fellow; 5 Professor of Epidemiology, Warwick Medical School, University of WarwickCoventry, UK; 6 Professor of Infectious Disease Epidemiology, School of Life Sciences, University of WarwickCoventry, UK; 7 Co-Director, Oxford Clinical Trials Research Unit, University of OxfordOxford, UK; 8 Professor of Rehabilitation, Clinical Trials Unit, University of WarwickCoventry, UK

**Keywords:** medical informatics, patient-centred care, person-centred medicine

## Abstract

**Rationale, aims and objectives:**

Classification of patients with back pain in order to inform treatments is a long-standing aim in medicine. We used latent class analysis (LCA) to classify patients with low back pain and investigate whether different classes responded differently to a cognitive behavioural intervention. The objective was to provide additional guidance on the use of cognitive behavioural therapy to both patients and clinicians.

**Method:**

We used data from 407 participants from the full study population of 701 with complete data at baseline for the variables the intervention was designed to affect and complete data at 12 months for important outcomes. Patients were classified using LCA, and a link between class membership and outcome was investigated. For comparison, the latent class partition was compared with a commonly used classification system called Subgroups for Targeted Treatment (STarT).

**Results:**

Of the relatively parsimonious models tested for association between class membership and outcome, an association was only found with one model which had three classes. For the trial participants who received the intervention, there was an association between class membership and outcome, but not for those who did not receive the intervention. However, we were unable to detect an effect on outcome from interaction between class membership and the intervention. The results from the comparative classification system were similar.

**Conclusion:**

We were able to classify the trial participants based on psychosocial baseline scores relevant to the intervention. An association between class membership and outcome was identified for those people receiving the intervention, but not those in the control group. However, we were not able to identify outcome associations for individual classes and so predict outcome in order to aid clinical decision making. For this cohort of patients, the STarT system was as successful, but not superior.

## Introduction

Almost everyone experiences non-specific low back pain sometime during their lifetime [1]. Each year in the UK, about one-third of the population experiences back pain and of this 20% consult their GPs about it [2]. There are a number of interventions known to be effective for non-specific low back pain including exercise programmes, manual therapy, acupuncture [2] and a cognitive behavioural approach [3]. However, these interventions have small effects when averaging cross the full population. This has prompted researchers to try to identify ways of classifying those seeking treatment so that patients can be matched to interventions in order to maximize treatment effects [4]. Many different classifications have been developed [5] but there is little consensus on their use [6]. For non-specific low back pain of greater than 6 weeks duration, the UK National Institute of Health and Clinical Excellence has suggested that patient preference should guide the choice of treatment from a range of effective interventions [2]. This study aimed to investigate LCA to classify patients and consider whether different groups responded differently to a cognitive behavioural intervention. The results could provide additional guidance for patients and clinicians as whether or not to consider the use of a cognitive behavioural approach [3].

We undertook secondary analysis of data from a multi-centred randomized controlled trial (RCT) of a primary care-based cognitive behavioural program for low back pain (the Back Skills Training Trial – ‘BeST’) [3]. The cognitive behavioural approach [3] targeted modifiable health behaviours and beliefs, for which evidence exists that they contribute to low back pain becoming chronic and disabling. The intervention targeted: activity levels, catastrophizing, fear avoidance, and coping skills. It comprised an assessment followed by six group sessions, which tackled these behaviours and beliefs, but did not target social factors such as educational level, work-related risk factors and job satisfaction. The control group received the internationally accepted best practice recommended for primary care which is to promote physical activity, prescribe analgesia and encourage a positive outlook. The trial found a positive effect of the intervention [3]. Pre-specified subgroup analysis of trial data found an interaction between baseline fear avoidance [7] and outcome (reduced disability) [8] with a larger treatment effect for those who were not fear avoidant at baseline, those whose back pain was at least very troublesome and those with a longer duration of back pain. This finding could be explained by the lack of effect on this group of patients of the treatment received in the control arm group of the trial [3]. In post hoc analysis by the trial team, no reliable evidence was found of a moderating effect of treatment outcome by baseline variables, although there was a suggestion that being younger and currently working moderated the treatment effect. In that post hoc analysis, back pain troublesomeness and fear avoidance had no moderating effects [9].

For our analyses, we used a latent class model to explore whether measures of a combination of modifiable beliefs and behaviours, amenable to clinical assessment and targeted by the cognitive behavioural approach, can be used to classify individuals with low back pain into different classes. LCA is a statistical technique that uses multivariate categorical data, to identify classes of similar individuals, the characteristics of the group and the probability of group membership. LCA is a probabilistic model because each class it identifies is characterized by a pattern of conditional probabilities that indicate the likelihood that variables take on certain values. The aim of latent class modelling is to obtain the number and definition of groups that best account for the dependencies between observed variables. We then assessed the impact on back pain-related disability, of class membership, and of the interaction between class membership and the intervention. There are many other widely used methods of classification, such as decision trees, random forests and support vector machines, which could be used for classification. The results of using artificial neural networks are reported elsewhere [10]. However, the purpose of this paper is not to conduct a comprehensive evaluation of analysis methods, but to try a probabilistic method that retains the clear interpretation of relationship between measured patient characteristics and prediction so vital in medical practice.

For comparison, the latent class approach to classifying patients was compared with the Subgroups for Targeted Treatment (STarT) back screening tool [11] which has been developed more recently. The STarT tool comprises a set of nine questions based on referred leg pain, co-morbid pain, disability, bothersomeness, catastrophizing, fear, anxiety and depression [12]. This tool classifies patients as low, medium or high risk of persistent disability, and treatment is tailored to each group: all patients were given advice and were shown a 15-minute educational video entitled Get Back Active20 and given the Back Book. Low-risk patients were only given this clinic session, medium-risk patients were referred for standardized physiotherapy to address symptoms and function, and high-risk patients were referred for psychologically informed physiotherapy to address physical symptoms and function, and also psychosocial obstacles to recovery. Our aim is to contribute to the development of evidence-based guidance for patients and clinicians when choosing between interventions known to be effective for low back pain.

## Methods

This secondary analysis was undertaken independently of the team that undertook the clinical trial. SL was the chief investigator of the trial and was involved in initial discussions about undertaking the analysis reported here and discussed the implications of the findings. During the trial, FG supervised the nested qualitative study only.

### Clinical trial setting and participants

Between April 2005 and April 2007, 701 adults with at least moderately troublesome low back pain of a minimum of 6 weeks duration [13] were recruited to the trial from 56 general practices in seven localities across England. Exclusion criteria were: aged <18 years, having factors associated with severe pathology, severe psychiatric or psychological disorders, and those who had had been managed previously in a cognitive behaviour programme [3]. Baseline measures were collected pre-randomization face to face. Outcome measures were collected by postal questionnaire. Random allocation was 2:1 for treatment: control arms of the trial.

### Data used in secondary analysis

We limited our analysis to the 407 participants of the 701 in the trial with complete data at baseline for the variables used for LCA and at 12 months for measuring outcome. We included those variables that were potentially modifiable by the intervention, and that could be assessed by a clinician in daily practice. These had been included in the trial data collection to provide indications as to why the treatment had or had not been effective [3]. The variables we chose included the fear avoidance beliefs questionnaire (items 2–5) [7] and the pain self-efficacy score [14] as they assess factors directly addressed by the intervention. We included the Hospital Anxiety and Depression Scores (HADS) [15] because challenging unhelpful thoughts, pacing, goal setting and relaxation are approaches used for the treatment of depression and anxiety [16], and back pain can become worse in the presence of depression [17]. We included question 7 of the SF12 [18] ‘During the past 4 weeks, how much of the time has your physical health or emotional problems interfered with your social activities (like visiting with friends, relatives, etc.)?’ to provide a single measure of the social impact of back pain as the intervention encouraged increased activity, and also included troublesomeness of back pain (moderately; very; extremely) as the intervention taught coping skills which might reduce troublesomeness. Scores were calculated according to questionnaire manuals.

HADS were split according to their standard interpretation; scores less than 7 were classed as not depressed or anxious, 7–10 borderline, and greater than 10 depressed or anxious [15]. The trial interpreted fear avoidance as follows: less than 14 not fear avoidant and 14 and over was fear avoidant, so we adopted these categories. No standard categorization exists for the pain self-efficacy score; we categorized into the following groups (0–20, 21–30 and 31–60) to differentiate between very low self-efficacy and low self-efficacy which could be clinically important, but disregarded the difference between high and very high self-efficacy.

We used the categorical outcome of a three-point change on the Roland–Morris Disability Questionnaire (RMQ) [19],[20], and calculated the change between baseline and 12 months (a decrease in score indicates decreased disability). Participants who scored 3 or less on the RMQ at baseline and also at 12 months were included in the improved group. There were no patients with a score of 22 or above (maximum disability score 24) at baseline or 12 months. Our adjusted models used age, gender and employment status.

### Statistical analysis

Polytomous variable LCA was implemented in R statistical software package poLCA [21] using both expectation-maximization and Newton–Raphson algorithms to fit finite mixture models. Two 3, 4, 5 and 6 class models were fitted separately and compared using Akaike Information Criterion (AIC) [22] and the Bayesian Information Criterion (BIC) [23].

Data from participants in both intervention and control arms of the trial were included in the analysis. Subjects were allocated to classes based on the maximum posterior probabilities of belonging to each class. Fisher's exact test was used to assess any association between class membership and outcome for the intervention group and for the control group. Logistic regression was used to explore whether class membership predicted improvement in back pain disability as defined.

The predictive ability of the logistic regression using only class membership to predict outcome was tested using 10-fold cross-validation [24]. This was compared with a simple logistic regression using all the variables used to construct the classes as predictors which was also tested using 10-fold cross-validation.

The patients in the BeST trial did not complete the STarT questionnaire, but there were sufficient data collected for us to construct STarT scores retrospectively. The BeST trial exclusion criteria would have eliminated anyone who responded positively to the first two questions on STarT. We selected available data to complete the remaining items in the STarT questionnaire as follows: STarT item 3 and RMQ question 17, STarT item 4 and RMQ question 9, Start item 5 and fear avoidance STarT item 6 and HADS question 5, STarT item 7 and pain self-efficacy, STarT item 8 and HADS question 2, STarT item 9 and troublesomeness. We calculated the STarT scores for each BeST patient according to the STarT tool instructions, and so obtained a second categorization of the BeST patients into three groups for comparison with the LCA classification. We tested the STarT group allocation for association with outcome using logistic regression and analysis of deviance as for the classes derived from LCA.

## Results

### Missing data

Of the 701 participants in the trial, 407 provided complete data for the variables used for determining class membership and outcome. Table 1 provides details of the missing. There were no missing values for age and gender but two cases of the 407 did not have data on employment status and were omitted when fitting the adjusted model.

**Table 1:** Counts of missing data (*n* = 701), with case and item missingness by variable, and accumulated sequentially

Variable	Complete cases (%)	Missing (%)		Accumulated (%)
		Cases	Items	
Fear avoidance	662 (94.4)	5 (0.7)	34 (4.9)	662 (94.4)
Anxiety	686 (97.9)	2 (0.3)	13 (1.8)	648 (92.4)
Depression	693 (98.9)	2 (0.3)	6 (0.8)	645 (92.0)
SF12 question 7*	689 (98.3)	12 (1.7)	–	635 (90.6)
Pain self-efficacy	676 (96.4)	3 (0.4)	22 (3.1)	620 (88.4)
Troublesomeness of back pain*	638 (91.0)	63 (9.0)	–	567 (80.9)
RMQ at baseline	700 (99.9)	1 (0.1)	0 (0.0)	567 (80.9)
RMQ 12 months	498 (71.0)	203 (29.0)	0 (0.0)	407 (58.1)

* Single-item score.

Of the 407 cases, 281 (69%) were in the intervention arm (60.0% of total in intervention arm of trial) and 126 (31%) in the control arm of the trial (54.1% of total in control arm of trial), which is in line with the 2:1 allocation to treatment.

### Latent class models and association with outcome for intervention and control patients

Models were fitted with 2, 3, 4, 5 and 6 classes. The model with the lowest BIC was the 3 class model and the model with the lowest AIC was the 4 class model (see Table 2).

**Table 2:** BIC and AIC for latent class models with 2–6 classes

Number of classes	2	3	4	5	6
BIC	4336.3	4304.6	4357.0	4426.3	4503.2
AIC	4211.9	4116.2	4104.5	4109.5	4122.3

AIC, Akaike Information Criterion; BIC, Bayesian Information Criterion.

There was an association between outcome and class for the 3 class model (Fisher's exact test *P* = 0.05) for those receiving the intervention, but not for the 2, 4, 5 or 6 class model. There was no association between outcome and class for those in the control arm of the trial for any model. Further analysis focused on the three-class model.

### Characteristics of classes in three-class model

Table 3 presents the characteristics of the members of each of the three classes and for all cases included in the LCA.

**Table 3:** Characteristics of the three classes: variables used to determine class membership; outcome variable; variables added in adjusted model

Variables used for identifying classes	Class I *n* = 213 (52.3%)	Class II *n* = 51 (12.5%)	Class III *n* = 143 (35.2%)	All *n* = 407 (100%)
SF12 Q 7*				
1	0	8	1	9
2	2	28	15	45
3	19	11	101	131
4	61	2	13	76
5	131	2	13	146
HADS anxiety†				
Not anxious	157	1	19	177
Borderline	43	1	79	123
Clinically anxious	13	49	45	107
HADS depression†				
Not depressed	206	0	51	257
Borderline	7	16	92	115
Clinically depressed	0	35	0	35
Troublesomeness of back pain‡				
Not troublesome	3	0	0	3
Moderately	123	11	61	195
Very	80	16	63	159
Extremely	7	24	19	50
Fear avoidance§				
Fear avoidant	76	44	91	211
Not fear avoidant	137	7	52	196
Pain self-efficacy¶				
Very low	3	19	4	26
Low	2	25	34	61
Moderate or higher	208	7	105	320
Outcome variables				
RMQ improvement**	90	18	73	181
No RMQ improvement**	123	33	70	226
Mean RMQ†† at baseline	6.38	13.86	9.64	8.46
Mean RMQ†† at 12 months	4.27	12.16	7.21	6
Variables added in adjusted model				
Age (years)				
Under 40	36 (16.9)	8 (15.7)	31 (21.7)	75 (18.4)
40–49	45 (21.2)	13 (25.5)	30 (21.0)	88 (21.7)
50–59	48 (22.5)	16 (31.4)	31 (21.7)	95 (23.3)
60–69	55 (25.8)	12 (23.5)	36 (25.1)	103 (25.3)
70 and over	29 (13.6)	2 (3.9)	15 (10.5)	46 (11.3)
Gender				
Male	99 (46.5)	15 (29.4)	47 (32.9)	161 (39.6)
Female	114 (53.5)	36 (70.6)	96 (67.1)	246 (60.4)
Employment status				
Working	128 (60.1)	16 (31.4)	65 (45.5)	209 (51.4)
Not working	84 (39.4)	34 (66.7)	78 (54.5)	196 (48.2)
Missing	1 (0.5)	1 (1.9)	0	2 (0.4)

* SF12 question 7 ‘How much of the time does your pain interfere with social activities?’ 1 = all of the time, 2 = most of the time, 3 = some of the time, 4 = a little of the time, 5 = none of the time.

† Hospital Anxiety and Depression Scores (HADS): <7 not depressed or anxious; 7–10 borderline; >10 depressed or anxious.

‡ Troublesomeness was used to screen patients prior to recruitment to exclude those without troublesome back pain.

§ Fear avoidance: <14 not fear avoidant; ≥14 fear avoidant.

¶ Pain self-efficacy: very low 0–20; low 21–30; moderate and higher 31–60.

** Three-point reduction on Roland–Morris scale or who scored 3 or less on the Roland–Morris scale at baseline and also at 12 month.

†† Roland–Morris Disability Questionnaire. RMQ score takes integer values 0–24, with lower score indicating less disability.

Most members of class I found their back pain did not interfere with social activities, they were not anxious or depressed, their back pain was moderately troublesome, they were not fear avoidant, and they had moderate or higher self-efficacy.

Most members of class II found their back pain interfered with social activities, they were anxious and depressed, their back pain was very or extremely troublesome, they were fear avoidant, and they had low or very low self-efficacy.

Most members of class III found their back pain interfered with social activities some of the time, they were borderline anxious and borderline depressed, their back pain was moderately or very troublesome, they were fear avoidant, and they had moderate or higher self-efficacy.

### Prediction of improvement with intervention for certain class of patients

Table 4 shows the percentage of each class in each arm of the trial and the number and percentage of patients in each arm of the trial who did and did not improve, divided into the three classes. Of those receiving the intervention, in class III a higher proportion improved than did not improve. In class I a higher proportion improved but the difference was not as marked as in class III, and in class II a higher proportion did not improve than improved.

**Table 4:** Participants who experienced improvement in back pain in each class of the three-class model

	Received intervention		
	Number that improved (%)		% of class
	No	Yes	
Excluded	167 (89.3)	20 (10.7)	63.6
Class I	73 (52.5)	66 (47.5)	65
Class II	25 (61.0)	16 (39.0)	80
Class III	41 (40.6)	60 (59.4)	71
Total	139 (49.4)	142 (50.5)	

	Control group		
	Number that improved (%)		% of class
	No	Yes	
Excluded	94 (87.9)	13 (12.1)	36.4
Class I	50 (67.6)	24 (32.4)	35
Class II	8 (80.0)	2 (20.0)	20
Class III	29 (69.8)	13 (30.2)	29
Total	87 (69.0)	39 (31.0)	

(Improved = three-point reduction on Roland–Morris scale or who scored 3 or less on the Roland–Morris scale at baseline and also at 12 months. Excluded: because one or more variables of interest were missing).

Logistic regression adjusted for age, gender and employment [9] indicated a predictive effect of improving with treatment for those in work (see Table 5).

**Table 5:** Logistic regression models to predict recovery from back pain: unadjusted and adjusted models

Variable	Class and treatment model			Adjusted model†		
	Coefficient	Standard error	*P*-value	Coefficient	Standard error	*P*-value
Intercept	−1.39	0.79	0.08#	−1.46	0.98	0.14
Cognitive behavioural approach (treatment)	0.94	0.85	0.27	0.78	1.09	0.47
Class I	0.65	0.82	0.43	1.08	0.91	0.23
Class II	0			0		
Class III	0.58	0.86	0.49	0.84	0.89	0.35
Treatment* Class I	−0.31	0.90	0.74	−0.82	0.99	0.41
Treatment* Class II	0			0		
Treatment* Class III	0.24	0.93	0.79	−0.0009	0.98	0.99
Treatment* work	–	–	–	1.48	0.62	0.02*

† Adjusted for age, gender and employment status.

Significance codes

# significant at α = 0.1

* significant at α = 0.05.

The error rate for the logistic regression using only class to predict outcome was 39.8% (sensitivity 0.59, specificity 0.61), and for the ordinary logistic regression the error rate was 41.5% (sensitivity 0.54, specificity 0.61).

We tested the STarT group membership for association with outcome in an identical fashion as for the classes derived from LCA. Both sets of results were remarkably similar. The medium-risk group was the smallest group, containing 20% of the patients. There were no differences between STarT groups in respect of the spread patient ages or gender. The low-risk group contained twice as many working patients as not working patients and this proportion was reversed for the high-risk group. In the medium-risk group, the numbers were even.

Logistic regression showed a strong association between treatment and outcome, but no association between membership of any of the STarT groups and outcome. The analysis of deviance showed a strong association between treatment and outcome and STarT group and outcome, but no significant interactions. A chi-square test between STarT group and outcome showed a strong association for the intervention patients but no association for control patients. Unlike the LCA, using the STarT groups did not pick out work status as significant; work is known to be an effect modifier [9].

## Discussion

We were able to classify the trial participants using a probabilistic model based on psychosocial baseline scores relevant to the intervention. Of the parsimonious models tested for association between class membership and outcome, an association was only found with one model which had three classes. For the trial participants who received the intervention, there was an association between class membership and outcome, but not for those who did not receive the intervention. However, we were unable to detect an effect on outcome from interaction between class membership and receiving the intervention or not.

The STarT tool, a popular, alternative, tree-based method, was employed for comparison and was found to perform equally well in most respects, but not better on this cohort of patients. In fact, our analysis using the STarT groups did not identify work status as an effect modifier, whereas the same analysis using the latent classes did. There remains a need for decision support for clinicians in allocating individual patients with non-specific low back pain to the most appropriate of the array of interventions that have been shown to be effective when outcome is averaged over all patients.

### Strengths and limitations of the study

By limiting our choice of data for analysis to that with direct relevance to the intervention mechanisms, we reduced the danger of identifying classes with no relevance to the question of deciding who might benefit from the intervention [4]. LCA can only be used with a complete data set. For linear models, there are methods for multiple imputation of missing values and pooling of model coefficients based on robust theory and which can quantify the uncertainty in the model coefficients, but these are not readily available for LCA. A clinical trials data set is relatively small for use with subgrouping [25]. A further limitation is that sensitivity to change is known to vary across the RMQ scale with better sensitivity in the middle range compared with the low and high ranges [26],[27]. Our analysis confirms the well-known association between employment status and back pain outcome [28].

### Classifying back pain to improve treatment outcome

A systematic review of the role of classification in low back pain published in 2011 [29] identified three types of classification: to classify by diagnosis, to describe prognosis, and considering treatment response, as in our study. The review identified 28 classification systems of which five were in the treatment-based category where treatment was tailored to the classification of the individual patient. In all five, classification was done by the clinician and based wholly or in part on observations about pain location and change with movement. With movement, two had been subject to a clinical trial and for only one of these was there some evidence of effectiveness (rated as insufficient).

Our study differs by including variables based on patient self-report and capturing a range of variables that influence the experience of back pain, rather than focusing only on the pain. In the RCT, high-risk patients received support for psychosocial barriers to recovery in addition to the physiotherapy received by the medium-risk group. All groups in the intervention arm received advice. There was a larger improvement in RMQ scores in the intervention group at 4 and 12 months, and the cost was lower [30]. The STarT tool was evaluated with an RCT involving 329 patients and a cohort study of 410. Our study is similar to the STarT study although without the rigour of being an RCT.

Unlike the pre-specified subgroup analysis and the post hoc analysis on the same data set as our study [3],[9], our LCA suggests there are classes of patients which benefit most from the intervention. This suggests a benefit from the use of LCA that is able to capture the non-linear relationships between variables. We have shown that the error rate using the classes as predictors of treatment response in a logistic regression is lower than using the variables on which the classes were built in a logistic regression, showing that classes add value to predictions of outcome. Moreover, while the specificity was the same for both regression models, the sensitivity was superior in the case of the model using the classes as predictors. Although we have shown a clear association between class and outcome for those who received the intervention, we have not been able to show a particular class that had had a statistically significant difference to treatment response. During the data cleaning, the algorithm was run on several versions of the data which varied by a few cases showing the optimal classes, class assignment, and association between class membership and outcome in the intervention group were robust. However, change in a few cases made a difference as to whether outcome was influenced by interaction between class membership and intervention. This suggests that the sample size, after excluding the missing data, was not large enough to be robust to individual patient effects, reducing statistical power of the study; there might be an effect that is too small to detect. Conversely, if the size of the effect of class membership exists but is small, then it is unlikely to have an important clinical use.

### Future use of LCA and the classification of back pain

A larger data set would enable greater use of methods such as LCA for classification of back pain such as the repository of individual data from back pain clinical trials [9]. Confidence in the finding that class membership influences the likely effect of the intervention would be increased if it was possible to detect the effect of interaction between class membership and treatment. Missing data remains a problem.

Combining variables relevant to back pain treatment for identifying classes results in a new qualitative assessment of the patient–class membership. This is somewhat similar to the patient-centred approach used by clinicians, which takes account of the whole person including their context and relationships [31]. However, our approach is restricted to considering factors which the intervention was designed to tackle. There is evidence that a clinician's assessment of a patient is influenced by the potential management routes available [32]. In contrast to this multidimensional classification used at baseline in our study, the outcome assessment consisted of a single measure of back pain disability. There is also evidence that patients can change qualitatively even if their scores on an outcome measure do not change [3],[33]. If a range of data had been collected at follow-up, the outcome assessment could also be qualitative and multidimensional.

Using LCA increases the complexity of assessments, as non-linear relationships between variables are captured. Using the same classification as at baseline, it might be possible to identify individuals that change class over time. This idea is supported by a small longitudinal interview study that included people with back pain, recruited in a similar way to the study reported here [34]. The categories they identified captured the emergent dynamic of individuals in relation to their back pain – emerging from the interaction of many different, not necessarily all acknowledged, factors [35]. Categorizing using a small range of variables, as here, gives a simpler qualitative assessment in which all components are measureable.

Classification post hoc to an intervention trial to seek categorizations that influenced outcome of intervention is an extremely attractive technique, but will always require RCTs to confirm that the associations found are genuine causal relationships rather than spurious, random associations. Nonetheless, the application of the same techniques to multiple data sets (i.e. multiple trials) would impart greater confidence that such associations are genuine. The main outcome from such techniques at the moment is to encourage consideration of variables (and meta-variables) that cannot be directly or easily be measured, and their potential for predicting outcomes.
